# Molecular Mechanisms of T Cells Activation by Dendritic Cells in Autoimmune Diseases

**DOI:** 10.3389/fphar.2018.00642

**Published:** 2018-06-26

**Authors:** Yu Tai, Qingtong Wang, Heinrich Korner, Lingling Zhang, Wei Wei

**Affiliations:** ^1^Key Laboratory of Anti-inflammatory and Immune Medicine, Ministry of Education, Anti-inflammatory Immune Drugs Collaborative Innovation Center, Institute of Clinical Pharmacology, Anhui Medical University, Hefei, China; ^2^Menzies Institute for Medical Research, Hobart, TAS, Australia

**Keywords:** T cell, dendritic cells, activation, autoimmune diseases, immunological synapse, biological agents

## Abstract

The interaction between T cell and dendritic cells (DCs) that leads to T cell activation affects the progression of the immune response including autoimmune diseases. Antigen presentation on immune cell surface, formation of an immunological synapse (IS), and specific identification of complex by T cells including two activating signals are necessary steps that lead to T cell activation. The formation of stimulatory IS involves the inclusion of costimulatory molecules, such as ICAM-1/LFA-1 and CD28/B7-1, and so on. Some fusion proteins and monoclonal antibodies targeting costimulatory molecules have been developed and approved to treat autoimmune diseases, including rheumatoid arthritis (RA), systemic lupus erythematosus (SLE), multiple sclerosis (MS), type I diabetes (T1D), inflammatory bowel disease (IBD), and psoriasis. These biological agents, including CTLA-4- and LFA-3-Ig, anti-CD3 monoclonal antibody, could prevent the successful engagement of DCs by T cell with significant efficacy and safety profile. In this article, we reviewed the molecular mechanisms of T cell activation during the interaction between T cells and DCs, and summarized some biological agents that target costimulatory molecules involved in the regulation of T cell activation.

## Introduction

Various organ specific autoimmune diseases are mediated by an imbalance of T cell subsets, e.g., an absence of regulatory T cells, or tissue injury driven by pathological T helper cell responses. Examples are rheumatoid arthritis (RA), systemic lupus erythematosus (SLE), multiple sclerosis (MS), type I diabetes (T1D), and inflammatory bowel disease (IBD) (Fletcher et al., 2010; Burmester et al., 2014; Geem et al., 2015; Suarez-Fueyo et al., 2016; Pugliese, 2017). In an inflammatory environment, autoreactive T cells are activated initially by dendritic cells (DCs). Like macrophages and B cells, DCs are professional antigen-presenting cells (APCs). However, DCs have the unique property of inducing the differentiation of naïve CD4^+^ T cells into helper and effector T cells with a unique combination of abilities, which allows DCs to effectively process antigen, strongly express costimulatory molecules, secrete cytokines, and migrate to tissues or lymphoid organs to prime T cells (Steinman, 2007). Therefore, DCs emerged as critical players in the initiation and development of immune response.

In identifying pathogen-associated cues, DCs undergo a series of functional changes known as maturation. Mature DCs present antigenic peptides in the context of major histocompatibility complex (MHC) class II to the T cell receptor and express co-stimulatory molecules CD40 and B7. Mature DCs are characterized by the production of cytokines, such as IL-12, and by the expression of homing receptors, such as CCR7, which directs the migration of DCs into the T-cell regions of secondary lymphoid organs. Together these changes enable DCs to effectively activate naïve T cells. At the same time, DCs induce the expression of the corresponding costimulatory molecules of CD40L, CD28, on T cells. Mature DCs promote naïve T cells differentiate into Th1, Th2, Th17, or Treg cells in a stimulus-dependent manner by secreting cytokines. In a mouse model of EAE *in vivo* and *in vitro*, DCs which express aberrant P38 can promote the differentiation of Th17 cells by inducing the secretion of IL-17 and the expression of IL-23 receptors (IL-23R) (Huang et al., 2012). Detection of mature DCs producing large quantities of IL-12 and IL-23 strongly supports the notion that DCs play a key role in autoimmune diseases by promoting a deleterious imbalance between Th1, Th2, and Th17 cells (Lebre et al., 2008; Tournadre et al., 2012; Segura et al., 2013). The majority of DCs exist in the inflammatory synovia fluid of RA patients, expressing CD1a and secreting IL-23 (Segura et al., 2013). Furthermore, DCs with a gene deletion of interleukin 1 receptor related kinase M (IRAK-M) show strong antigen presenting ability, resulting in the abnormal activity of diabetogenic T cells and autoimmune reaction *in vitro* and the rapid onset of T1D *in vivo* in immunodeficient NOD mice when cotransferred with diabetogenic T cells (Tan Q. et al., 2014).

DCs include immunogenicity DCs and tolerogenic DCs according to function. Interactions between tolerogenic DCs and CD4^+^Foxp3^+^ regulatory T cells (Tregs) play a critical role in maintaining peripheral tolerance and preventing activation of T cells (Audiger et al., 2017). Peripheral tolerance is associated with a high activity of Tregs and a reduced inflammatory profile of Th cells (Min et al., 2006; Li et al., 2008). CD4^+^ Treg in both the spleen and lymph node help to maintain tolerogenic status of DCs through the expression of CTLA-4 in mice (Wing et al., 2008).

DCs from different location exert different functions. Plasmacytoid DCs secrete large amounts of type I interferons (such as IFN-alpha and IFN-beta) after identification of the viruses through TLR7 and TLR9, which are located in intracellular compartments (Gilliet et al., 2008). The central role of plasmacytoid DC in autoimmune diseases is emphasized by its association with type I-interferon signal. Type I interferons produced by plasmacytoid DC from human PBMCs also supports IL-17 secretion and Th17 responses (Lombardi et al., 2009). Furthermore, human plasmacytoid DCs enhance thymic Treg expansion and generation of peripheral Treg through the production of indoleamine 2, 3-dioxygenase (IDO) and the expression of programmed death-ligand 1 (PD-L1) *in vivo* and *in vitro* (Chen et al., 2008; Amarnath et al., 2011; Creusot et al., 2014). Lymphoid-resident DCs rapidly extracts antigens from lymph and blood for presentation to T cells (Sixt et al., 2005). In particular, CD205^+^ DCs in the spleen of mice are able to induce the tolerance of CD4^+^ T cell under suboptimal activation conditions (Yamazaki et al., 2008).

The interaction between T cells and DC leads to the formation of immunological synapse (IS) and is maintained by highly expressing adhesion molecules (LFA-1, LFA-3, ICAM-1, ICAM-2), cytokines and chemokines (Lee et al., 2002; Tseng et al., 2008). In this article, we reviewed the molecular mechanism of T cells activation by DCs and immunotherapy targeting T cell activation in autoimmune diseases.

## Molecular Mechanisms of T Cell Activation by DCs

There are three stages during T cells activation by DCs, namely antigen presenting, antigen recognition of T cells and two signals formation. In addition, IS formation between T cells and DCs plays an important role in T cell activation.

### Antigen Presenting

Germline encoded pattern recognition receptors (PPR) specific for pathogen-associated patterns (PAM) are present on immature DCs. An engagement of these membrane-bound receptors trigger a maturation of DCs and lead to an up-regulation of costimulatory molecules (Kabelitz and Medzhitov, 2007). Mature DCs in mice express chemokine receptor 7 (CCR7) and begin to migrate into regional lymph nodes after an encounter with antigen (Ritter et al., 2004).

For a presentation with MHC class II, antigen is degraded by DCs to a suitable length (approximately 12 amino acids) utilizing proteasomes in the endogenous pathway. These antigenic peptides bind to specific grooves in the MHC class II molecules (Jones et al., 2006). Peptide-MHC II complexes are formed in the rough endoplasmatic reticulum and transported to the cell surface for presentation (Vyas et al., 2008; Neefjes et al., 2011). At the local draining lymph node, DC present complexes of processed peptides together with MHC class II to naïve CD4^+^ T cells which bind to this combination with their TCR and initiate signaling. The peptide binding to MHC class I and the subsequent presentation to CD8^+^ T cells is similar in many aspects and will not be discussed in detail. Overall, antigen presentation with MHC class II and MHC class I are mainly two modes for DCs.

A third mode of antigen presentation utilizing the conserved non-classical MHC class I molecule CD1 plays an important role in microbial infection and the immune response to lipid antigens (Shao et al., 2005; Barral and Brenner, 2007). CD1 has 30% homology with MHC class I molecules, and there are main five types of CD1 in humans, termed CD1a-e (Barral and Brenner, 2007). Probably the best studied member of the CD1 family is CD1d which presents predominantly lipids to CD1-restricted T cells that have a limited repertoire of TCR and have been termed Natural Killer (Kang et al., 2014). Although interferon (IFN)-gamma secretion by CD1-restricted T cells during infection had been shown before, the function of CD1 restricted T cells was not entirely understood for a long time (Gilleron et al., 2004). Only recently, it was demonstrated that the expression of human class I CD1 molecules in transgenic mice caused a rapid response of CD1-restricted T cells after re-exposure to antigen, suggesting a protective effect of CD1-restricted T cells (Felio et al., 2009). Natural sebum can be used as a headless antigen and presented to autoreactive T cells through CD1a (de Jong et al., 2010). In addition, researchers have found a group of highly conserved T cells in tuberculosis (TB) patients. These conserved T cells could recognize specifically mycobacterial antigens presented by CD1b (Van Rhijn et al., 2013). These findings suggest that the antigen-presenting molecule CD1 plays an important role during special antigen presentation.

### Antigen Recognition of T Cells

T cell receptor (TCR) on T cells not only identify peptide-MHC complexes derived from host cells infected by pathogens, but also recognize similar structures derived from healthy host cells, so called autoantigens. The specificity of the TCR for antigen is located in the V segment, which is composed of N-terminal of two TCR polypeptides (Govers et al., 2010). Both V alpha and V beta have 3 hypervariable regions and are also known as the complementarity determining region, namely CDR1, CDR2, and CDR3. CDR3 is a largest hypervariable region and directly determines the antigen binding specificity of TCR (Feng et al., 2015). The TCR identifies simultaneously the entire complex of antigenic peptide and MHC molecule as a first step in T cell activation (Reiser et al., 2000). The comparison between the TCR conformation and the conformation of TCR bound to the peptide-MHC complex indicates that CDR3 region undergoes a large conformational transition in order to obtain a diagonal position that allows the binding to peptide-MHC complex (Garcia, 2012). When the V segment of the TCR identifies an antigen/MHC complex, the TCR heterodimer deliver the activation signal to the cell nucleus through the constant transmembrane components of the CD3 complex. (Schamel et al., 2005). Therefore, TCR signaling has a key role in determining T cell fate. TCR-peptide-MHC complexes appear to support a model of physical specificity between TCR germline V regions and MHC.

### Two Signals Are Necessary for Activation of Naïve T Cells

**FIGURE 1:**
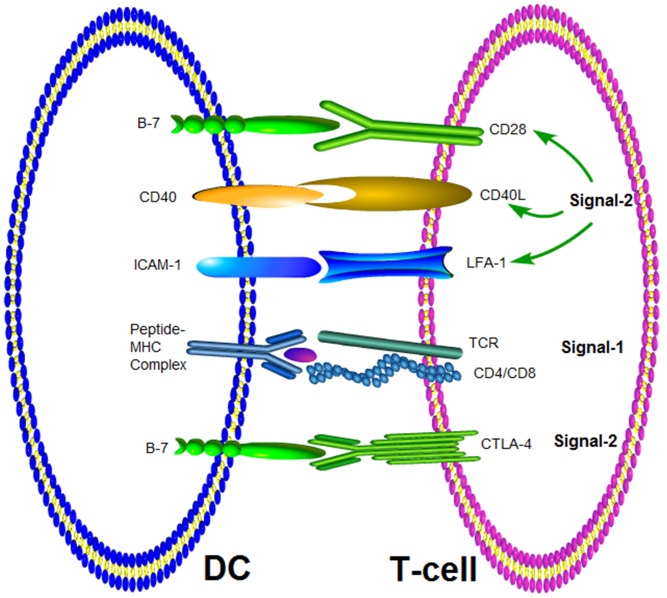
Molecular interactions at the interface of T-cell and APC. Signal-1 is provided by the interaction between TCR and the MHC-peptide complex. The co-stimulatory signal (Signal-2) can be delivered by different pairs of molecules.

Naïve T cell activation by DCs requires two signals, termed signal-1 and signal-2 (Anderson and Siahaan, 2003). Signal-1 is equivalent to the binding of TCR to peptide-MHC complex (Garg et al., 2010; Vesely et al., 2011; Manikwar et al., 2012). Signal-2 requires the interaction of costimulatory molecules at the interface between DCs and T cells (B7/CD28, LFA-1/ICAM-1 and ICAM2, CD2/LFA-3) (**Figure 1**). The combination of TCR and peptide-MHC complex (signal 1) will lead to phosphorylation of the immunoreceptor tyrosine-based activation motif (ITAM) on CD3, which is closely adjacent to TCR by Lck kinase (Rossy et al., 2013). T cells receive signal-1 when the activation cascade leads to signaling through multiple TCR for several hours (Frauwirth and Thompson, 2004). This sustained signaling is necessary for the effective activation of signal transduction pathways that lead to the activation of nuclear transcription factors. The clustering of TCR in IS at the interface between T cells and DC is necessary for continuous signal transduction and will be discussed in more detail later. Signal-2 was initialized by the interaction of costimulatory molecules expressed DCs and T cells. Positive signals, such as CD28/B7-1 (CD80) and CD28/B7-2 (CD86), and negative signals, such as CTLA-4/CD80 and CTLA-4/CD86 have been identified (Huang et al., 2012; Manikwar et al., 2012). As mentioned above pairs of costimulatory molecules (CD80/CD28, LFA-1/ICAM-1, or ICAM2, CD2/LFA-3) provide signal 2. The activation signal of these costimulatory molecules is delivered to T cells via the ITAM of the cytoplasmic domain, which enhances the TCR response to antigen (Acuto et al., 2008). It was two signals model for T-cell activation. T cells could be activated in simultaneously receiving signal-1 from T-cell recognition of antigen and signal-2 from costimulatory molecular. However, it was an off signal to T cells in only receiving signal-1, and T cells would translate into tolerance, clone incompetent or deletion.

CD28/CD80 was a pair of co-stimulatory molecules that mediated and enhanced immune responses, but these molecules were not directly involved in memory immune responses (Kopf et al., 2000). Furthermore, the co-stimulatory signal of CD28 was related mainly to initiating interaction between DCs and T cells. The CD28/CD80 signal activate T cells to express multiple other costimulatory molecules, these costimulatory molecules control the balance of immune response and the stability of internal environment. The interaction between cytotoxic T lymphocytes (CTL) and Th or Th and B cells rests on other costimulatory molecules, such as OX40 (CD134), inducible T-cell costimulator (ICOS) (Bansal-Pakala et al., 2004). The costimulatory molecule 4-1BB and its ligand 4-1BBL can control adaptive immunity (Lee et al., 2008). Treg cells up-regulate the expression of 4-1BB in response to IL-2 and suppressed T cell proliferation. At the same time, the synergy of 4-1BB and CD28 signal can affect cell polarization, and promote Th0 cells differentiation into Th1 cells which are characterized by the production of IFN-gamma (Elpek et al., 2007).

CTLA-4 (CD152) is homologous to CD28 and also expressed on activated T cells, but the cytoplasmic domain of CTLA-4 has an immunoreceptor tyrosine-based inhibitory motif (ITIM) (Topalian et al., 2015). Therefore, CTLA-4 binds to CD80 in competition with CD28 with an affinity that is 20 times higher than CD28/CD80 and can send an inhibitory signal to activated T cells through its ITIM motif to restore the balance of immune response (Pentcheva-Hoang et al., 2004; Vogel et al., 2015). CTLA-4 activates protein tyrosine phosphatase (PTP) through the ITIM structure and inhibits T cell activation signal transduction, leading to a negative regulation of T cell activation (Chemnitz et al., 2004). Additionally, CTLA-4 inhibits the expression of IL-2 receptor alpha chain, IL-2 secretion and IL-2 mRNA accumulation, also resulting in an inhibition of the activation of T cells in preclinical mouse models (Hannani et al., 2015). Hence, costimulatory signals mediated by costimulatory molecules, including positive signals and negative signals, play important role in regulating interaction between T cell and DCs and maintaining the balance of immune response.

### IS Formation Plays an Important Role in T Cell Activation

IS play an important role in T cells activation, and IS formation involves a variety of costimulatory molecules, such as ICAM-1/LFA-1, CD28/B7-1, and so on (Schwartz et al., 2002).

### Formation Mechanisms of IS

In the process of T cell and DC interaction, a variety of transmembrane molecules accumulate in a “raft” structure that is rich in sphingomyelin and cholesterol, and are clustered at the interface of T cell and DC contact. This special “raft” structure has been termed IS. Before the formation of IS, T cells form pseudopods in search of peptide MHC complexes on APCs. After the initial contact the formation of IS is a dynamic process that has been described to depend on a planar lipid bilayer. IS formation includes three phases: (i) The first stage is the connection of TCR and peptide MHC complex. The adhesion molecules such as LFA-1/ICAM-1 and CD2/LFA-3 are recruited to the nascent rafts (Barreiro et al., 2007); (ii) The second stage has been termed the peptide MHC complex transfer stage. In the early stages of IS formation, TCR- peptide MHC complex is transported to the central region of IS, while LFA-1/ICAM-1 is transferred to the peripheral region to form mature IS; (iii) The third stage is the formation of a stable contact region at the interface of T cell and antigen presenting cell. In this stage, the super molecular structure of a mature IS can be maintained for 1 h, while PKC theta, Bcl10, IKK beta are recruited to IS by cytoskeleton changes (Dustin, 2005).

### Molecular Structure of IS

**FIGURE 2:**
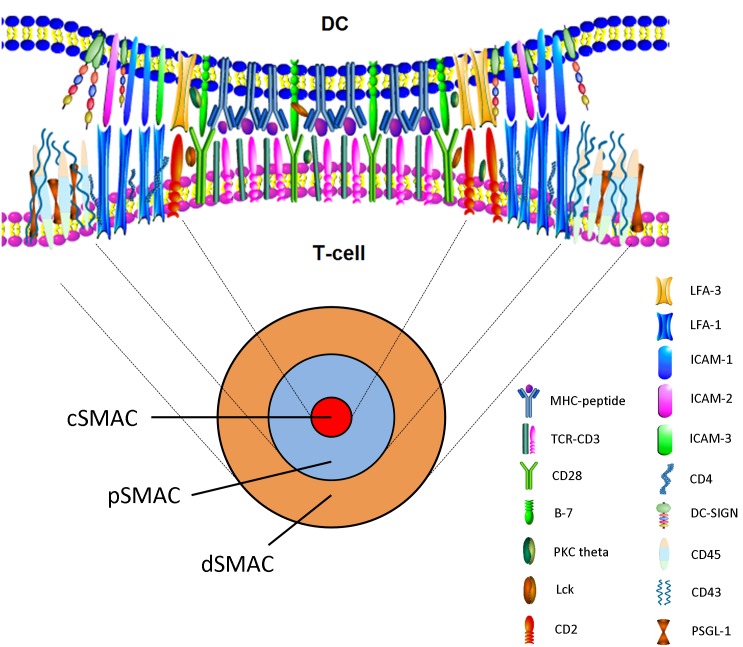
En face view of the IS with cSMAC, pSMAC, and dSMAC. Active reorganization via cytoskeleton-directed movements gives rise to the cSMAC. The mature synapse with the cSMAC, the pSMAC, and the dSMAC is observed after T-cell–APC contact.

The molecular structure of IS include three areas, namely central supermolecular activation cluster (cSMAC), peripheral SMAC (pSMAC), and distal SMAC (dSMAC). The molecules in cSMAC area mainly includes TCR- peptide MHC complex, CD3, CD28, and signal transduction molecules such as PKC theta and Lck (Valitutti, 2008). Adhesion molecules such as LFA-1/ICAM-1 or DC-SIGN surround pSMAC area (Dustin, 2005). CD2/LFA-3 is located between cSMAC and pSMAC, and CD43, CD45, and PSGL-1 are located at dSMAC. The number of TCRs in cSMAC is only double that of in pSMAC, but the number of LFA in pSMAC is almost 6 times that of in cSMAC. In fact, cSMAC and pSMAC do not show obvious boundaries. Although cSMAC and pSMAC can be maintained for several hours, the numbers of receptors and molecules in IS are changed dynamically (Dustin, 2005). CD45 is a unique molecule that is transferred to dSMAC from cSMAC, which may be related to the activation of Lck at the stage of early IS formation (Grigorian et al., 2009). cSMAC take part in the reuse and degradation of TCRs, which down-regulate the TCR and attenuate signals (**Figure 2**). Thus it can be seen that the molecular structure of IS was very complex involving in many molecules and signals, which take part in IS formation through interaction and dynamically balance.

### Factors That Influence IS Formation

TCR signaling is necessary for the maintenance of IS. TCR-microclusters (TCR-MCs) are formed immediately after the TCR on T cells adheres to peptide MHC complex including many signal molecules such as CD3, SLP-76, TCR, and ZAP-70 (Campi et al., 2005; Saito and Yokosuka, 2006). TCR-MCs are the activation site of initial signal. TCR stimulation, calcium influx, tyrosine activation occur before the formation of cSMAC. TCR-MCs are continuously produced, even after IS formation (Barda-Saad et al., 2005). TCR is rapidly internalized about 5 min after the exposure to DC, but TCR stimulation will continue for several hours, which results in the activation signal in the peripheral MCs rather than cSMAC. Blocking TCR-peptide–MHC complex within 10 hours results in IS dissolution, decreasing the level of calcium influx and causes cell separation. These findings indicate that the maintenance of TCR signaling is necessary for the maintenance of IS and the full activation of T cells.

CD4 molecule could promote IS formation. CD4 can improve the sensitivity of T cells to antigens and can accumulate Lck to the center region of IS after the initial exposure of DCs to T cells. CD4-deficient T cells have a reduced proliferative response to antigen stimulation and the effect of IS formation is also significantly reduced. The cells expressing CD4 or displaying a peptide MHC complex have a strong binding, suggesting that CD4 is not only an auxiliary receptor but also contributes to cell adhesion. The actual lipid raft is the key components of a functional IS. The accumulation of lipid rafts was observed at cSMAC, indicating that the formation of IS was accompanied by the movement of lipid rafts to cSMAC and gathered on the contact interface of cells (Henel et al., 2006). Lck and LAT are linked to lipid rafts by deacylation. Other signaling molecules such as PLC gamma, SLP76, and Vav are transferred to IS by binding to phosphorylated tyrosine on LAT (Phee et al., 2005; Braiman et al., 2006; Soares et al., 2013). CTLA-4 limits the accumulation of lipid rafts, thereby inhibiting T cell proliferation and cytokine secretion (Teft et al., 2006). Actin movement is associated with the transportation of cytoskeleton and can be blocked by the myosin inhibitor butanedione monoxime (van der Honing et al., 2010).

Overall, TCR signaling, CD4 molecule, lipid raft, PLC gamma and CTLA-4, and so on not only involve in IS information but also modulate IS information. Any abnormal signals or the imbalance among molecules would lead to abnormal T cell activation in autoimmune diseases. These molecules might be new drug targets, and it would offer new therapy strategies through developing new drugs targeting above molecules.

## Immunotherapy Targeting T Cells Activating in Autoimmune Diseases

Detailed insights into the molecular mechanisms of the interaction between T cells and DCs are helpful to design immunotherapy strategies that target T cell activation in autoimmune diseases. At present, some biological agents, such as CTLA-4Ig (Abatacept), Anti CD3 monoclonal antibody, LFA-3 Ig fusion protein (Alefacept) that target co-stimulation molecules on T cell have been developed and approved to treat autoimmune diseases.

### CTLA-4Ig Modulates Co-stimulatory Signals and Inhibits T Cell Activation

The recombinant fusion protein, CTLA-4Ig (Abatacept) that comprises the extracellular domain of human CTLA4 and a fragment of Fc domain of human IgG1 belongs to a new type of selective co-stimulatory modulators. Abatacept prevents complement fixation and modulates the necessary co-stimulatory signal for T cell activation. Furthermore, it binds to CD80 and CD86, thus competing with CD28 and reducing T cell activation (Cutolo and Nadler, 2013; Keating, 2013). The fusion protein affects multiple downstream pathways through modulation of upstream events of T cells activation. Additionally, Abatacept inhibits T-cell proliferation and the secretion of IFN-gamma, IL-1, IL-6, and TNF-alpha (Koenders et al., 2012; Whitfield et al., 2017).

As therapy, Abatacept is mainly used in RA treatment (Genovese et al., 2005; Dorner et al., 2010). It has been proven to be efficient, safe, and tolerable in combination with methotrexate (MTX) in clinical trials with RA patients when the response to MTX was inadequate (Kremer et al., 2005). In Europe, Abatacept is approved for the treatment of patients with highly active and progressive RA, who have never received MTX treatment. It is also approved for the treatment of patients with moderate to severe active RA, who have shown inadequate responses in previous therapies with at least one conventional disease-modifying antirheumatic drug (cDMARDs) such as MTX or a TNF inhibitor. In phase III clinical trials, intravenous or subcutaneous injection regimens of Abatacept were beneficial for RA symptoms, disease activity, structural damage progression and physical function of the joint. In a long-term follow-up, the efficacy could be shown to be maintained. The drug-free remission rates following discontinuation of all RA treatment were significantly higher after treatment of Abatacept plus methotrexate than of methotrexate alone. Intravenous and subcutaneous injections of Abatacept were generally well tolerated and showed low immunogenicity (Blair and Deeks, 2017). Previous studies using synovial tissue from RA patients treated with Abatacept found the inhibition of B-cell proliferation and down regulation of the expression of B-cell markers (Buch et al., 2009).

Abatacept was also used to treat lupus nephritis by inhibiting CD28 engagement on T cells and plasma cells (Bahlis et al., 2007). This mechanistic rationale is strongly supported by the studies in SLE murine models, in which treatment with Abatacept or other forms of CTLA4-Ig have been shown to arrest and even reverse established lupus nephritis. Treatment with Abatacept induced remission by binding to CD80 on renal podocytes in patients with focal segmental glomerulosclerosis (Yu et al., 2013; Group, 2014).

### Anti-CD3 mAbs by Induce Anergy and Apoptosis of Activated T Cells

Anti CD3 monoclonal antibodies are an immunosuppressant. Muromonab-CD3 is a murine IgG2, which specifically binds to CD3 on T cells and blocks proliferation and function of T cells. Tolerance induction by anti-CD3 mAbs is related to the induction of Tregs that control pathogenic autoimmune responses preferentially by inducing anergy or apoptosis in activated T cells while ignoring Tregs (You et al., 2008; Penaranda et al., 2011). Consequently, anti-CD3 mAb therapy is associated with an increase in number and function of Treg and regulatory cytokines such as TGF-beta and IL-10. The heterogeneity of TCR expression by different T-cell subsets might explain the differential effect of anti-CD3 mAb on effector, regulatory or naïve T cells (Valle et al., 2015). At the same time anti-CD3 mAb-induced signaling through the CD3/TCR complex can render the T cell anergic or trigger apoptosis. Various studies have shown that anti-CD3 mAbs effectively treat chronic inflammatory and autoimmune diseases, such as IBD, T1D and MS. Intravenous administration of anti-CD3 mAb has been successfully tested in numerous animal models of autoimmunity, including the experimental autoimmune encephalomyelitis (EAE) model of MS, diabetic NOD mice, TNP-KLH induced colitis (a model of IBD) and collagen-induced arthritis (Kohm et al., 2005; Chatenoud and Bluestone, 2007; Notley et al., 2010; Wu et al., 2010).

Biological agents targeting CD3 include teplizumab, otelixizumab, and visilizumab. It has been observed that administration of otelixizumab, teplizumab, or visilizumab result in positive clinical responses (Keymeulen et al., 2005, 2010; Plevy et al., 2007). Otelixizumab and teplizumab were foremost tested in T1D patients, while visilizumab and foralumab were mainly studied in IBD (Yu et al., 2008; Daifotis et al., 2013). In clinical trials, the tolerogenic activity of humanized anti-CD3 mAb (visilizumab) in T1D was found to be excellent. In a second Phase I/II trial, teplizumab improved insulin production and metabolic control in patients with recent onset T1D. A phase II trial that assessed the safety and efficacy of visilizumab in patients with severe corticosteroid-refractory ulcerative colitis had promising results (Plevy et al., 2007). In general, non-FcR binding anti-CD3 mAb are promising models for treatment of autoimmune and inflammatory diseases (Herold et al., 2003).

### LFA-3 Ig (Alefacept) and Anti-LFA-1 Antibody (Efalizumab) Inhibit CD2 Signaling in T Cells

It had been demonstrated that an LFA-3 Ig fusion protein (Alefacept) could reduce psoriasis lesions (Nickoloff and Nestle, 2004). Alefacept competes with LFA-3 for binding to CD2 on T cells and efficiently interferes with LFA-3/CD2 binding, consequently stopping T cell activation. Furthermore, the Ig part of Alefacept binds to immunoglobulin receptor Fc-gamma-RIII on the surface of natural killer cells and some T cell subpopulations inducing apoptosis of memory T cell subgroups (da Silva et al., 2002; Rigby et al., 2015). Finally, administered intramuscularly or intravenously Alefacept inhibits T cell activation and proliferation, and induces the apoptosis of memory-effector (CD45RO^+^) T cells (Konig et al., 2016).

In psoriasis the presence of LFA-1 in IS is very important. A separate anti-LFA-1 antibody (Efalizumab) has been shown to block adhesive interaction in the treatment of psoriasis. The antibody reduced skin lesions by blocking the adhesion molecule on T cells and was well tolerated and resulted in significant improvement in patients with moderate to severe plaque psoriasis (Papp et al., 2006).

## Conclusion

In summary, the interaction between T cells and DCs involves in the pathogenesis of autoimmune disease. Autoreactive T cells are activated by autoantigens presented by DCs during the interaction between T and DC (Tan T. et al., 2014). The underlying molecular mechanisms of T cell activation by DCs have been well understood. Three stages during T cells activation by DCs, including antigen presenting, antigen recognition of T cells, and two signals formation have been investigated in great detail. T cells could be activated in two signals model by simultaneously receiving signal-1 from T-cell recognition of antigen and signal-2 from costimulatory molecular. In addition, IS formation between T cells and DCs plays an important role in T cell activation. cSMAC, pSMAC, and dSMAC form the molecular structure of IS. IS molecular structure is very complex involving in a variety of molecules and signals, which take part in IS formation through interaction and dynamically balance.

Understanding the molecular mechanisms of the interaction between T cells and DCs is helpful to discover new drug targets and design immunotherapy strategies that target T cell activation in autoimmune diseases. At present, some recombinant fusion protein and monoclonal antibodies targeting costimulatory molecules, such as CTLA-4- and LFA-3-Ig, anti-CD3 monoclonal antibody, and so on have been developed and approved to treat autoimmune diseases, such as RA, SLE, IBD, MS, and psoriasis. These biological drugs show a significant efficacy and have a high safety profile. More biological agents that modulate T cell activation will be developed based on a better understanding of the molecular mechanisms of T cell activation in the near future.

## Author Contributions

YT and QW collected data and wrote this paper. HK and LZ revised the article. WW designed the work.

## Conflict of Interest Statement

The authors declare that the research was conducted in the absence of any commercial or financial relationships that could be construed as a potential conflict of interest.
